# The YY1/miR-548t-5p/CXCL11 signaling axis regulates cell proliferation and metastasis in human pancreatic cancer

**DOI:** 10.1038/s41419-020-2475-3

**Published:** 2020-04-27

**Authors:** Wan-Li Ge, Qun Chen, Ling-Dong Meng, Xu-Min Huang, Guo-dong Shi, Qing-Qing Zong, Peng Shen, Yi-Chao Lu, Yi-Han Zhang, Yi Miao, Jing-Jing Zhang, Kui-Rong Jiang

**Affiliations:** 1 Pancreas Center, The First Affiliated Hospital of Nanjing Medical University, Nanjing, China; 2 Pancreas Institute, Nanjing Medical University, Nanjing, China; 3 Ultrasonography, The First Affiliated Hospital of Nanjing Medical University, Nanjing, China

**Keywords:** Pancreatic cancer, miRNAs, Transcriptional regulatory elements

## Abstract

Pancreatic cancer (PC) is a malignant tumor with a poor prognosis and high mortality. However, the biological role of miR-548t-5p in PC has not been reported. In this study, we found that miR-548t-5p expression was significantly decreased in PC tissues compared with adjacent tissues, and that low miR-548t-5p expression was associated with malignant PC behavior. In addition, high miR-548t-5p expression inhibited the proliferation, migration, and invasion of PC cell lines. Regarding the molecular mechanism, the luciferase reporter gene, chromatin immunoprecipitation (ChIP), and functional recovery assays revealed that YY1 binds to the miR-548t-5p promoter and positively regulates the expression and function of miR-548t-5p. miR-548t-5p also directly regulates CXCL11 to inhibit its expression. A high level of CXCL11 was associated with worse Tumor Node Metastasis (TNM) staging in patients with PC, enhancing proliferation and metastasis in PC cells. Our study shows that the YY1/miR-548t-5p/CXCL11 axis plays an important role in PC and provides a new potential candidate for the treatment of PC.

## Introduction

Pancreatic ductal adenocarcinoma (PDAC) is the fourth leading cause of cancer death in the United States and the relative 5-year survival rate is only ∼9%^1^. Surgery is the only way to cure pancreatic cancer (PC), but only 5–20% of patients have an opportunity to receive surgery when diagnosed; even so, the median survival of surgically resected patients is <20 months and only ∼20% of all resected patients survive up to 5 years^2–4^. The effects of radiotherapy and chemotherapy are also limited^5,6^. Therefore, it is urgent to delve into the molecular mechanisms to find new therapies for PC. Over the past years, many mRNAs relative to the development and progression of PC have been studied^6,7^. However, the mechanism of noncoding RNA, such as microRNA (miRNA), remain largely unknown.

miRNA is a type of short-chain noncoding small RNA, which can bind to the 3′-untranslated region (3′-UTR) of target gene mRNA by base complementary pairing, causing degradation of mRNA^8^. Previous studies have shown that abnormal miRNA expression often affects PC cell proliferation, apoptosis, migration, invasion, drug resistance, and other functions, leading to tumor progression^9,10^. The has-miR-548 family currently has 69 members. Many of them have been found to play important roles in the tumorigenesis and development of certain cancers^11–13^. However, the function of miR-548t-5p has not been well studied and no one has demonstrated its function. Our previous chromatin immunoprecipitation (ChIP) sequencing found that the transcription regulator Yin Yang-1 (YY1) binds to the promoter region of miR-548t-5p. At the same time, its highly abnormal expression in PC attracted our interest.

In this study, we investigated the role of miR-548t-5p and found that it is a tumor suppressor gene. We also found that the transcription factor YY1 positively regulates miR-548t-5p expression and the YY1/ miR-548t-5p/CXCL11 axis inhibits PC development.

## Materials and methods

### Cell culture

PC cell lines (BXPC-3, CFPAC-1, COLO-357, MIAPACA-2, and PANC-1) and the normal human pancreatic ductal cell line hTERT-HPNE (HPNE) were purchased from Shanghai Cell Bank (Shanghai, China). Cells were cultured in Dulbecco’s modified Eagle’s medium (DMEM) (Life Technologies) containing 10% fetal bovine serum (FBS) (Wisent, Inc., Montreal, QC, Canada), 1% glutamine (Sigma), and 1% penicillin/streptomycin (Life Technologies) at 37 °C in a humidified incubator with 95% air and 5% CO_2_.

### Tissue specimens and tissue microarrays

Fifty pairs of tumorous and adjacent nontumorous human pancreatic tissues (2015–2016) were collected from patients who had not undergone chemotherapy or radiation therapy before tumor excision. The 50 patients were followed up regularly until 1 November 2018. A tissue microarray (TMA) containing another 95 pairs of tumorous and adjacent nontumorous human pancreatic tissues (2016–2017) was constructed by Servicebio (Wuhan, China). All 145 patients were from the First Affiliated Hospital of Nanjing Medical University. Before specimen collection, written informed consent was obtained from the patients or their relatives and the study was approved by the Ethics Committee of the First Affiliated Hospital of Nanjing Medical University. Each sample was snap-frozen in liquid nitrogen immediately upon removal. Histopathological diagnoses and differentiation were confirmed by two experienced pathologists. Tumor Node Metastasis (TNM) staging was based on the 8th edition of the American Joint Committee on Cancer guidelines^14^.

### Preparation of stable cells

YY1-overexpression and YY1-knockdown lentiviruses were constructed by Sunbio Medical Biotechnology Co., Ltd (Shanghai, China). The structures of the YY1 lentiviruses have been described in a previous report^15^. LV3-hsa-miR-548t-5p mimic and control lentiviral vectors were constructed by GenePharma (Shanghai, China) and the constructs were verified by sequencing. PANC-1 and BXPC-3 cells at ∼40% confluence were infected with YY1-overexpression, YY1-knockdown, LV3-hsa-miR-548t-5p mimic lentiviral vectors, and the respective controls at an appropriate multiplicity of infection. Stable cell lines were selected by culturing in complete medium containing 5 µg/ml puromycin (Sigma). The effects of transfection were verified by quantitative reverse-transcription PCR (qRT-PCR) and western blotting.

### Transient transfection of oligonucleotides, siRNAs, and plasmids

Oligonucleotide miR-548t-5p-mimics (sense 5′-CAAAAGUGAUCGUGGUUUUUG-3′, antisense 5′-CAAAAACCACGAUCACUUUUG-3′), miR-548t-5p inhibitor (5′-UCUCAAAAACCACGAUCACUUU UG-3′), and their corresponding controls (m-NC for mimics, sense 5′-UUUGUACUACACAAAAGUACU G-3′, antisense 5′- CAGUACUUUUGUGUAGUACAAA-3′ and i-NC for inhibitor, 5′-CAGUACUUUU GUGUAGUACAAA-3′) were constructed by Ribobio (Guangzhou, China). CXCL11-overexpression and empty vector plasmids were purchased from Genecopoeia (Rockville, MD, USA). CXCL11-knockdown small interfering RNA (siRNA) (sense 5′-CUGUCUUUGCAUAGGCCCUTT-3′, antisense 5′-AGGGCCUAUGCAAAGACAGTT-3′) and controls (sense 5′-UUCUCCGAACGUGUCACGUTT-3′, antisense 5′-ACGUGACACGUUCGG AGAATT-3′) were synthesized by Biotend (Shanghai, China). Transient transfections were performed using Lipofectamine 3000 (Life Technologies) according to the manufacturer’s instruction. The expression of the target gene was determined by qRT-PCR and western blotting.

### RNA isolation and quantitative RT-PCR

Total RNA was extracted from the tissues and cells using TRIzol reagent (Life Technologies, Carlsbad, CA, USA) according to the manufacturer’s instructions. For mRNA detection, 500 ng of total RNA was reverse transcribed to cDNA in a final volume of 10 µL using a commercial kit (Bio-Rad, Hercules, CA, USA) according to the manufacturer’s instructions. For miRNA detection, specific primers were constructed according to the manufacturer’s instructions and qRT-PCR was performed using FastStart Universal SYBR Green Master Mix (Roche, Basel, Switzerland) and a 7500 Real-time PCR System (Applied Biosystems, Carlsbad, CA, USA). The relative expression of mRNA or miRNA was normalized to that of β-actin, 18S RNA, or U6 and it was calculated by the 2^−ΔΔCT^ method^16^. Each quantitative PCR was performed in triplicate and repeated independently three times. The primers are shown in Supplementary Table S1.

### Fluorescence in situ hybridization

Fluorescence in situ hybridization (FISH) assays were performed using a FISH Kit (RiboBio, Guangzhou, China). BXPC-3 and PANC-1 cells were washed with phosphate-buffered saline (PBS) and fixed in 4% formaldehyde for 10 min. The cells were then permeabilized with PBS containing 0.5% Triton X-100 at 4 °C for 5 min and washed with PBS three times for 5 min, followed by prehybridization at 37 °C for 30 min. The cells were hybridized at 37 °C overnight in the dark with an anti-miR-548t-5p, anti-U6, or anti-18S oligodeoxynucleotide probe, counterstained with DAPI (4′,6-diamidino-2-phenylindole) and imaged using a Nikon Eclipse Ti-E inverted fluorescence microscope (Nikon, Japan).

### MicroRNA in situ hybridization

To analyze miR-548t-5p expression in PC tissues, in situ hybridization (ISH) analysis was performed using a double digoxigenin (DIG)-labeled probe (sequence: 5′-DIG-AAAAACCACGATCACTTTTG-DIG-3′). Slides were deparaffined and rehydrated before incubation with proteinase K at 37 °C for 30 min and the slides were washed three times with PBS for 5 min. Hybridization solution containing the miR-548t-5p probe (concentration: 8 ng/µL) was added and hybridized overnight at 50 °C in an incubator. After hybridization, the slides were washed with 2× saline sodium citrate (SSC) for 10 min at 37 °C, twice with 1× SSC for 5 min at 37 °C, and with 0.5× SSC for 10 min at room temperature. Sealing serum was added at room temperature for 30 min; after removing the sealing solution, rat anti-digoxin-labeled alkaline phosphatase (anti-DIG-AP) was added and incubated at 37 °C for 40 min. Hybridization signals were visualized by 5-Bromo-4-Chloro-3-Indolyl Phosphate/nitro-blue tetrazolium and the nucleus was stained with a nuclear solid red dye solution. After rinsing with pure water and air drying, the sections were sealed with neutral gum. Semiquantitative scoring for ISH was evaluated according to the sum of the staining intensity (0 for absent, 1 for weak, 2 for moderate, and 3 for strong staining) and the proportion of positive cells (0 for <10%, 1 for 10 to <50%, and 2 for ≥50% of cells).

### Immunohistochemistry

Immunohistochemistry (IHC) was performed to determine the CXCL11 (Abcam, Cambridge, MA, USA, 1:50) and Ki-67 (Servicebio, China, 1:200) levels. In brief, the extracted tissues were fixed with formalin (4%) and embedded in paraffin. Next, 4 µm-thick paraffin-embedded tissue slices were cut and transferred to glass slides. The slides were deparaffinized in xylene, rehydrated in alcohol, and washed with tap water. Endogenous peroxidase activity was blocked by immersing the sections in 3% hydrogen peroxide. The sections were treated with 10 mmol/L sodium citrate buffer (pH 6.0) at 95 °C for 20 min for antigen retrieval, followed by a blocking step with 5% normal goat serum for 10 min at 37 °C. The slides were then incubated at 4 °C with primary antibodies overnight, followed by incubation with secondary antibodies for 30 min at 37 °C. Finally, diaminobenzidine was added for ∼5 min and hematoxylin was used for counterstaining. The histochemistry score (H-SCORE) was calculated to assess the expression level of CXCL11 based on the staining intensity and the positive cell ratio as follows: H-SCORE = (percentage of cells with weak staining × 1) + (percentage of cells with medium staining × 2) + (percentage of cells with strong staining × 3). The proliferation index was calculated according to the Ki-67 staining as follows: we generally selected areas with abundant tumor cells and many positive cells, selected ten high-power fields, calculated the ratio of positive cells to the total number of cells, and finally used the average value as the proliferation index.

### Cell counting kit-8 assay

Cell proliferation was detected using a Cell counting kit-8 (CCK-8) kit (Dojindo, Japan) according to the manufacturer’s instructions. A total of 2000 cells per well were inoculated with complete medium and cultured in 96-well plates for 5 days; 10 µL of CCK-8 reagent and 90 µL of complete medium were mixed and added to each well every 24 h and incubated at 37 °C for 2 h. Absorbance was assessed at 450 nm (A450) using a spectrophotometer.

### Colony formation assay

A total of 800 PC cells were plated in 6-well plates and cultured in complete medium for 16 days. The colonies were fixed with 4% paraformaldehyde for 15 min, stained with a 0.05% crystal violet solution for 30 min, and counted directly under a Zeiss microscope. Colonies consisting of more than 50 cells were counted.

### 5-Ethynyl-2’-deoxyuridine assay

An 5-Ethynyl-2’-deoxyuridine (EdU) assay kit was used to measure cell proliferation following the manufacturer’s instructions (RiboBio, China). A total of 8000 cells were seeded into 96-well plates and cultured for 48 h, after which 100 µl EdU medium (50 µM) was added to each well and incubated for 2 h. The cells were then fixed in 4% paraformaldehyde for 30 min, permeabilized with 0.5% Triton X-100 for 10 min, and incubated with 100 µL of 1× ApolloR reaction cocktail for 30 min followed by 100 µL of Hoechst 33342 for 30 min. Images were acquired under a Nikon microscope (Nikon, Japan). Each sample consisted of three duplicate wells, with three repeats.

### Wound-healing assay

A wound-healing assay was performed to evaluate cell migration ability. For the assay, 8 × 10^5^ cells were seeded per well into a six-well plate. After the cells grew to confluence, the monolayer was wounded with a 200 µL pipette tip and floating and detached cells were removed with PBS. The cells were then cultured in DMEM without serum to inhibit proliferation. Images were taken at 0 and 48 h and the area of the wound was measured with ImageJ software.

### Transwell assay

Transwell assays were performed to assess the migration and invasion abilities of the cells. For the migration experiment, 40,000 cells suspended in serum-free medium were added to the upper chamber and 10% FBS medium (750 µL) was added to the bottom chamber to induce cell migration to the other side of the membrane. After 24 h of culture, the chamber was removed and fixed with methanol for 30 min. The upper cells were wiped away with cotton swabs and then stained with a 1% crystal violet solution for 20 min. For the invasion experiment, Matrigel (BD Bioscience Pharmingen, USA) was added to the upper chamber according to the instructions. Except for culturing for 48 h, the treatment was the same as described above. Cells in five random fields of view under ×100 magnification were counted. All experiments were repeated three times.

### Flow cytometric analysis of the cell cycle and apoptosis

Flow cytometry (FCM) was performed to examine the cell cycle and apoptosis. The cell cycle was detected using a Cell Cycle Analysis Kit (Multi Sciences, China). The cells were digested, washed once, and re-suspended in 1 mL of PBS. The cell suspension was slowly added to 3 mL of precooled anhydrous ethanol (−20 °C) and fixed overnight at −20 °C. Before FCM detection, the cells were hydrated with 3 mL of PBS for 15 min and then stained with 1 mL of DNA staining solution containing 10 µL of permeabilization solution for 30 min at room temperature in the dark.

Apoptosis was detected using an Annexin V-fluorescein isothiocyaate (FITC) kit according to the manufacturer’s instructions (Multi Sciences, China). In brief, cells were collected and stained with Annexin V-FITC (50 µg/mL) and propidium iodide (10 µg/mL) in the dark for 5 min before being subjected to FCM (LSR, BD Biosciences).

### Western blotting

The PC cells were lysed with radioimmunoprecipitation assay lysis buffer and protein was extracted as described previously^15^; the protein concentrations were quantified using a DC protein assay kit (Bio-Rad, USA). The proteins were subjected to 8–12% SDS-polyacrylamide gel electrophoresis and transferred to a polyvinylidene difluoride membrane (Bio-Rad, USA)^17^. The membranes were then blocked with 5% non-fat milk in Tris-buffered saline containing 0.1% Tween (TBST) for 2 h at room temperature and incubated with primary antibodies overnight at 4 °C. After washing with TBST (15 min) three times, the membranes were incubated with horseradish peroxidase (HRP)-conjugated anti-mouse or anti-rabbit IgG secondary antibodies for 2 h at room temperature. The membranes were washed again and the protein expression levels were visualized with the Immobilon Western Chemilum HRP substrate (Merck Millipore, Darmstadt, Germany) using an enhanced chemiluminescence detection system. The primary antibodies against CXCL11 and vimentin were obtained from Abcam (Cambridge, MA, USA); the primary antibodies against E-cadherin and N-cadherin were obtained from Proteintech (Han Wu, China). Each blotting was repeated independently three times.

### Luciferase activity assay

For promoter analysis, the wild-type or mutant sequence of the miR-548t-5p promoter region in which the presumed YY1-binding site was 5′-CAACATGGATGA-3′ was constructed using the pGL3-Basic vector (Promega, Madison, WI, USA). To examine the relationship between YY1 and miR-548t-5p promoter activity, wild-type or mutant constructs of the miR-548t-5p reporter plasmid were cotransfected with YY1 or vector. The *Renilla* luciferase expression plasmid was used as a reference control. After transfection for 48 h, the luciferase activities were detected using dual luciferase reporter assays (Promega, E1910, WI, USA).

For a 3ʹ-UTR analysis, luciferase reporters carrying the WT (pMIR-REPORT-CXCL11-WT-3′-UTR) and mutated CXCL11 3ʹ-UTR (pMIR-REPORT-CXCL11-MUT-3′-UTR) were synthesized by Obio Technology (Shanghai, China). The reporter plasmids and miR-548t-5p-mimics were cotransfected into 293T cells using Lipofectamine 2000 (Invitrogen). The transfection method and the procedures were the same as those for the luciferase activity assay described above.

### Chromatin immunoprecipitation assay

ChIP assays were performed using an EZ ChIP kit (Millipore, Darmstadt, Germany) according to the manufacturer’s instructions. Lysates were incubated with antibodies against YY1 or normal mouse IgG, and qRT-PCR was performed to amplify the purified DNA fragment using SYBR Green Master Mix (Roche, Basel, Switzerland; 40 cycles). The primers used are as follows: forward primer, 5′-GCCTCTGCTTAAATCTAAGTTGTA-3′; reverse primer, 5′-TGAGAACATGCAATACTTGTCT-3′ (product length: 158 bp). The PCR products were analyzed using 2% gel electrophoresis.

### Digital gene expression sequencing

Six micrograms of total RNA was extracted from BXPC-3-miR-548t-5p mimic or BXPC-3-miR-548t-5p mimic NC cells. Quality and quantity analyses of total RNA, digital gene expression (DGE) library preparation, and sequencing were performed at Vazyme Biotech Co., Ltd (Nanjing, China). RNA with RNA integrity values > 7 was used to prepare RNA-sequencing libraries. After the acquisition of raw reads, quality control, and data filtering, paired-end reads were mapped to the human genome using the Tophat2 tool and the expression levels of the genes were determined using the Cufflinks tool (version 2.2.1). DGE analysis was performed with the cuffdiff function integrated into the Cufflinks tool. An absolute value of log2 ratio ≥ 1 and false discovery rate < 0.05 were applied as thresholds to judge the significance of the gene expression differences. DGE data are displayed by heatmaps and Venn plots.

### Bioinformatics

The Jaspar database was used to predict whether YY1 binds to the promoter of miR-548t-5p. Potential miRNA targets were predicted using microarray data and the three following publicly available databases: DIANA, miRDB, and TargetScan. Targets were selected only when they were positive according to all four analyses. The database the Cancer Genome Atlas (TCGA) was used to determine the effect of CXCL11 on overall survival.

### In vivo model

Four-week-old male, nude mice (BALB/cA-nu) were purchased from the Animal Center of Nanjing Medical University. All animal experiments were conducted according to animal protocols approved by Nanjing Medical University and the study was approved by the Ethics Committee of the First Affiliated Hospital of Nanjing Medical University. For the in vivo tumor growth study, animals were divided randomly into five groups (BXPC-3-YY1 short hairpin RNA (shRNA), BXPC-3-scrambled shRNA, BXPC-3-YY1-shRNA + miR-548t-5p mimic, BXPC-3-miR-548t-5p mimic, or BXPC-3-miR-548t-5p mimic NC) and each group had five mice. Cells (1 × 10^6^ cells/100 µL per flank) were injected subcutaneously into the flanks. The tumor sizes were measured every week for 30 days, and the formula (width^2^ × length)/2 was used to calculate the tumor volumes. For the in vivo tail vein tumor metastasis study, the animals were divided randomly into two groups (BXPC-3-miR-548t-5p mimic or BXPC-3-miR-548t-5p mimic NC). Cells (1 × 10^6^ cells/100 µL) were injected separately into the tail vein of each mouse. Four weeks later, the mice were killed and the lungs and livers were removed and fixed in 4% paraformaldehyde; deparaffinized sections were stained with hematoxylin-eosin (HE). The histomorphology of the tumor samples and extent of metastasis in the lungs and livers were evaluated.

### Statistical analysis

Statistical analysis in the current study was performed using GraphPad Prism (version 6.0) and SPSS software (version 22.0). Quantitative data are presented as the mean (±SD). Differences in the mean between two groups were analyzed by Student’s *t*-test. Pearson’s *χ*^2^-test was employed to analyze associations of miR-548t-5p or CXCL11 expression with clinicopathologic features. miR-548t-5p-YY1 or CXCL11-miR-548t-5p interaction tests were performed using linear regression models. The Kaplan–Meier test was applied to calculate survival rates and log-rank tests were used to examine differences in survival rates between two groups. Area calculations were performed with ImageJ. The data obtained using tumor models were analyzed by Fisher’s exact test. All statistical tests were two-tailed exact tests with *p* < 0.05 considered significant (*p < 0.05, **p < 0.01, ***p < 0.001 and ****p < 0.0001).

## Results

### miR-548t-5p is downregulated in PC tissues

**Fig. 1:**
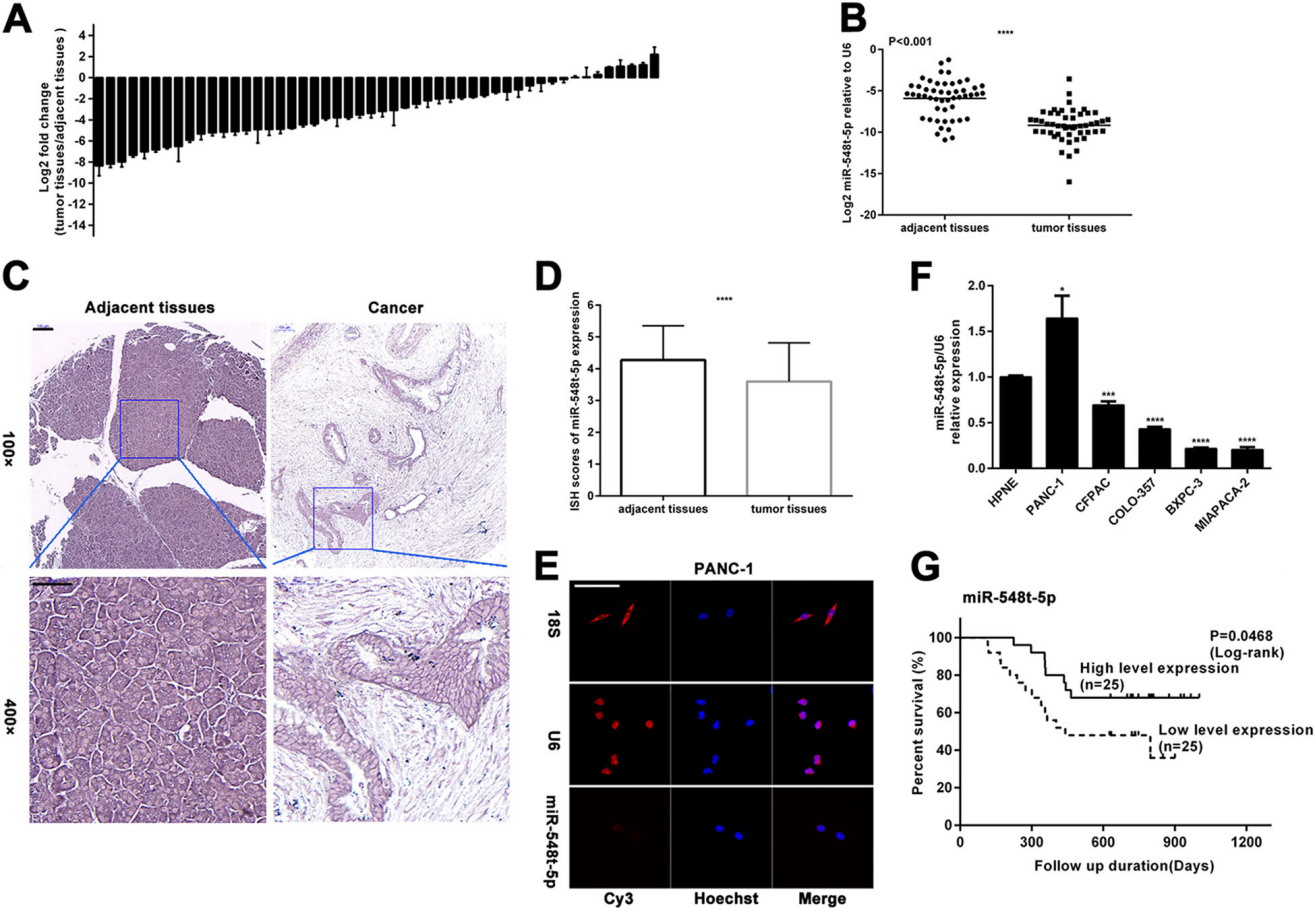
miR-548t-5p is downregulated in PC. **a** qRT-PCR analysis of miR-548t-5p expression in 50 pairs of human PC tissues and adjacent non-neoplastic tissues. **b** As analyzed by qRT-PCR, miR-548t-5p expression in PC tissues was significantly lower than that in the corresponding adjacent non-neoplastic tissues. **c** Representative ISH images of miR-548t-5p expression. Scale bar, 100 μm in ×100, 50 μm in ×400. **d** A histogram showing the ISH results of miR-548t-5p expression in the 95 pairs of human PC tissues and adjacent non-neoplastic tissues. **e** MicroRNA FISH showed that miR-548t-5p was mostly located in the cytoplasm. Magnification, ×400; scale bar, 50 μm. **f** miR-548t-5p expression in normal human pancreatic ductal cell line hTERT‐HPNE and PC cell lines BXPC-3, CFPAC-1, COLO-357, MIAPACA-2, and PANC-1 by qRT-PCR. **g** Kaplan–Meier curves for overall survival (OS) by miR-548t-5p expression. The data are presented as mean ± SD from three independent experiments. *P ≤ 0.05, **P ≤ 0.01, ***P ≤ 0.001, ****P ≤ 0.0001.

First, we examined the expression of miR-548t-5p in 50 pairs of human PC and adjacent non-neoplastic tissues by qRT-PCR. As shown in Fig. 1a, miR-548t-5p expression was downregulated in 42 of the 50 PC samples and it was reduced by up to 4-fold in 30 samples. Overall, miR-548t-5p expression was significantly lower in PC tissues than in adjacent non-neoplastic tissues (Fig. 1b). To confirm this finding, TMAs consisting of 95 pairs of PC and adjacent non-neoplastic tissues were used for ISH and, as shown in Fig. 1c, d, miR-548t-5p expression was downregulated in the PC tissues. To further explore the role of miR-548t-5p in PC, we determined the distribution of miR-548t-5p in PC cells by FISH and found miR-548t-5p to be localized mainly in the cytoplasm (Fig. 1e and Supplementary Fig. S1A). Furthermore, we examined miR-548t-5p expression in the normal human pancreatic ductal cell line hTERT-HPNE and PC cell lines (BXPC-3, CFPAC-1, COLO-357, MIAPACA-2, and PANC-1). As shown in Fig. 1f, the expression of miR-548t-5p was lower in CFPAC, COLO-357, BXPC-3, and MIAPACA-2 cells and higher in PANC-1 cells than in HPNE cells.

### Correlation between miR-548t-5p expression and pancreatic cancer

Fifty PC patients were included in the survival analysis. The cutoff value for low/high miR-548t-5p expression was determined by the median expression value based on qRT-PCR data. The Kaplan–Meier survival curves showed that patients with lower miR-548t-5p expression had lower overall postoperative survival (Fig. 1g, *p* = 0.0468).

In addition, the correlation between miR-548t-5p expression and the clinicopathological characteristics of PC patients was analyzed. As presented in Table 1, no significant association was observed between the miR-548t-5p expression levels and sex, age, tumor location, N stage, or serum carcinoembryonic antigen (CEA), but the miR-548t-5p expression levels did correlate negatively with the T stage, nerve infiltration, and serum CA199 values (*p* = 0.024, *p* = 0.024, and *p* = 0.001, respectively). The above results indicate that miR-548t-5p plays an important role in the genesis and development of PC, and that lower miR-548t-5p expression predicts poor survival in PDAC patients.

**Table 1:** Association of miR-548t-5p and CXCL11 expression with clinicopathological features of PDAC.

		miR-548t-5p expression			CXCL11 expression		
Variable	Group	High	Low	*P*-value	High	Low	*P*-value
Gender							
	Male	15	20	0.123	19	16	0.355
	Female	10	5		6	9	
Age (years)							
	<60	15	18	0.37	16	17	0.765
	≥60	10	7		9	8	
Location							
	Head	15	17		16	16	1
	Body/tail	10	8		9	9	
T stage							
	T1	10	3	0.024*	2	11	0.004*
	T2 or T3	15	22		23	14	
N stage							
	Absent	12	13	0.777	9	16	0.048*
	Present	13	12		16	9	
TNM stage							
	I	11	10	0.774	7	14	0.045*
	II–IV	14	15		18	11	
Nerve infiltration							
	Yes	15	22	0.024*	22	15	0.024*
	No	10	3		3	10	
Serum CA19-9 (kU/L)							
	≤37	14	3	0.001*	6	11	0.136
	>37	11	22		19	14	
Serum CEA (ug/L)							
	≤5	18	16	0.544	17	17	1
	>5	7	9		8	8	

*p < 0.05. Statistically significant difference.

### miR-548t-5p weakens proliferation of PC cells

**Fig. 2:**
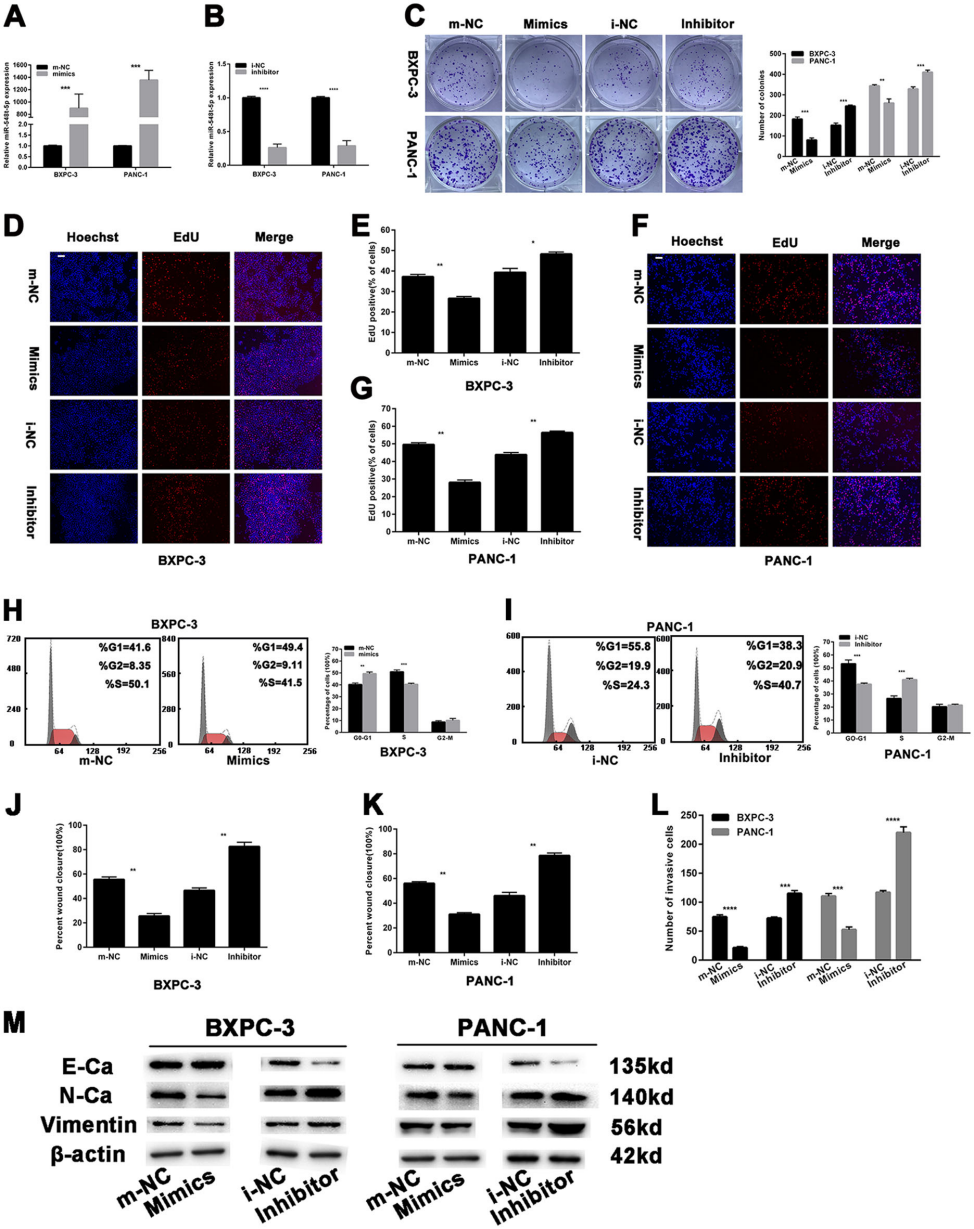
The effects of miR-548t-5p on PC cells proliferation, migration, and invasion. **a**, **b** Relative expression of miR-548t-5p by qRT-PCR in PC cells with miR-548t-5p-mimics and inhibitor. **c**–**g** The colony formation assay (**c**) and EdU assay (**d**–**g**) were performed to analyze the effects of miR-548t-5p-mimics or inhibitor on cell proliferation. Magnification, ×100; scale bar, 100 μm in **d**, **f**. **h**, **i** The flow cytometry assay analyzed the effect of miR-548t-5p on cell cycle status. **j**, **k** Wound-healing assays measured the effect of miR-548t-5p on PC cell migration ability. **l** Transwell assays measured the effect of miR-548t-5p on PC cell invasion ability. **m** EMT-related proteins were analyzed by western blotting assays in transfected PC cells. The data are presented as mean ± SD from three independent experiments. *P ≤ 0.05, **P ≤ 0.01, ***P ≤ 0.001, ****P ≤ 0.0001.

To elucidate the role of miR-548t-5p in PC development, BXPC-3 and PANC-1 cells were transfected with miR-548t-5p-mimics or inhibitor. The transfection efficiency of the cell lines (BXPC-3 mimics, BXPC-3 inhibitor, PANC-1 mimics, and PANC-1 inhibitor) was assessed by miRNA qRT-PCR (Fig. 2a, b). Colony formation assays showed fewer colonies of PANC-1 and BXPC-3 cells transfected with miR-548t-5p-mimics than control cells. In contrast, transfecting BXPC-3 and PANC-1 cells with the miR-548t-5p inhibitor had the opposite effect (Fig. 2c). An EdU assay was also performed to evaluate the effect of miR-548t-5p on proliferation. As depicted in Fig. 2d–g, the proportion of EdU-positive nuclei was significantly decreased in BXPC-3 and PANC-1 cells transfected with miR-548t-5p-mimics, whereas the miR-548t-5p inhibitor had the opposite effect.

Next, a flow cytometry assay was performed, revealing more G0/G1-phase and fewer S-phase BXPC-3 cells after miR-548t-5p mimic transfection; the opposite outcome was observed for PANC-1 cells transfected with the miR-548t-5p inhibitor (Fig. 2h, i). In addition, according to the FCM analysis of apoptosis, the BXPC-3 cells transfected with miR-548t-5p-mimics exhibited a higher rate of apoptosis than the NC cells (Supplementary Fig. S1B). In contrast, the miR-548t-5p inhibitor-treated PANC-1 cells displayed a lower rate of apoptosis than the NC cells. The above results reveal that miR-548t-5p inhibits the proliferation of PC cells.

### miR-548t-5p weakens the metastasis of PC cells

To determine whether miR-548t-5p has effects on cell migration and invasion, wound-healing and Transwell invasion assays were performed. As illustrated in Fig. 2j, k and Supplementary Fig. S2A, B, the miR-548t-5p-mimics markedly inhibited the migration of PANC-1 and BXPC-3 cells, whereas the miR-548t-5p inhibitor had the opposite effect. Moreover, when compared with the corresponding NC cells, fewer miR-548t-5p mimic-transfected BXPC-3 and PANC-1 cells invaded to the lower side of the membrane (Fig. 2l and Supplementary Fig. S2C). Conversely, more miR-548t-5p inhibitor-treated cells invaded to the lower side of the membrane. As epithelial-mesenchymal transition (EMT) is closely related to cell migration and invasion, we performed western blotting to examine the relationship between miR-548t-5p and EMT-related proteins. As shown in Fig. 2m, after miR-548t-5p overexpression, N-cadherin and vimentin were downregulated, whereas E-cadherin was upregulated. The opposite results were found for the miR-548t-5p-knockdown cell lines. These results suggest that EMT proteins are likely to be involved in the inhibitory effects of miR-548t-5p on PC cell metastasis. The above results demonstrate that miR-548t-5p inhibits the metastasis of PC cells.

### miR-548t-5p is positively regulated by the transcription factor YY1

**Fig. 3:**
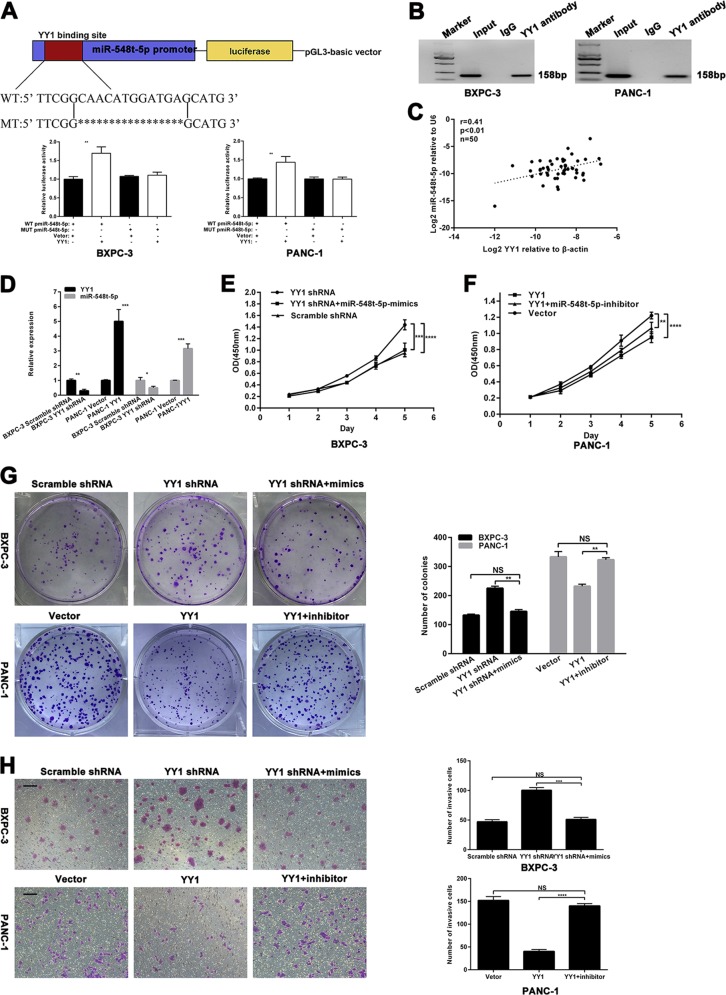
miR-548t-5p is regulated directly by the transcription factor YY1. **a** Schematic diagram of the luciferase reporter construct containing the human miR-548t-5p promoter and the mutant YY1 construct containing miR-548t-5p promoter in which the presumed YY1-binding site was mutated. Results revealed that YY1 directly bound to the promoter region of has-miR-548t-5p. **b** ChIP assays were performed in BXPC-3-YY1-OE and PANC-1-YY1-OE cells. Lane 1, DNA marker; lane 2, input DNA; lane 3, DNA from cells immunoprecipitated with normal rabbit IgG; lane 4, DNA from cells immunoprecipitated with anti-YY1 antibody. **c** In human PC tissues, miR-548t-5p was positively correlated with YY1 expression. **d** YY1 and miR-548t-5p expression levels in YY1-overexpressing or YY1-knockdown cells were measured by qRT-PCR. **e**–**g** CCK-8 and colony formation assays were performed to analyze proliferation in BXPC-3-YY1-knockdown cells transfected with miR-548t-5p-mimics and PANC-1-YY1-overexpression cells transfected with miR-548t-5p inhibitor. **h** Transwell assay was performed to analyze invasion in BXPC-3-YY1-knockdown cells transfected with miR-548t-5p-mimics and PANC-1-YY1-overexpression cells transfected with miR-548t-5p inhibitor. Magnification, ×100; scale bar, 100 μm. The data are presented as mean ± SD from three independent experiments. *P ≤ 0.05, **P ≤ 0.01, ***P ≤ 0.001, ****P ≤ 0.0001.

Based on our previous YY1 ChIP sequencing, we predicted that YY1 might bind to the promoter of miR-548t-5p and luciferase activity assays were performed to determine the relationship between YY1 and miR-548t-5p. As indicated in Fig. 3a, YY1 overexpression significantly increased fluorescence compared with the control conditions. Furthermore, ChIP assays revealed that YY1 binds directly to the promoter region of miR-548t-5p in vitro (Fig. 3b).

We next analyzed the expression of YY1 and miR-548t-5p in PC tissues by qRT-PCR and found that miR-548t-5p expression correlated positively with YY1 mRNA expression (Fig. 3c; *p* < 0.01, *r* = 0.41). Furthermore, YY1 knockdown in BXPC-3 cells and YY1 overexpression in PANC-1 cells led to decreased and increased miR-548t-5p expression, respectively (Fig. 3d).

To assess whether the effects of YY1 on PC cells are indeed mediated by miR-548t-5p, CCK-8, colony formation, and Transwell assays were performed. The results showed that the proliferation and invasion capacities inhibited by YY1 overexpression were recovered by downregulating miR-548t-5p, and that miR-548t-5p overexpression had the opposite effect on cells with YY1 knockdown (Fig. 3e–h).

### YY1 inhibits the development of PC by upregulating miR-548t-5p in vivo

**Fig. 4:**
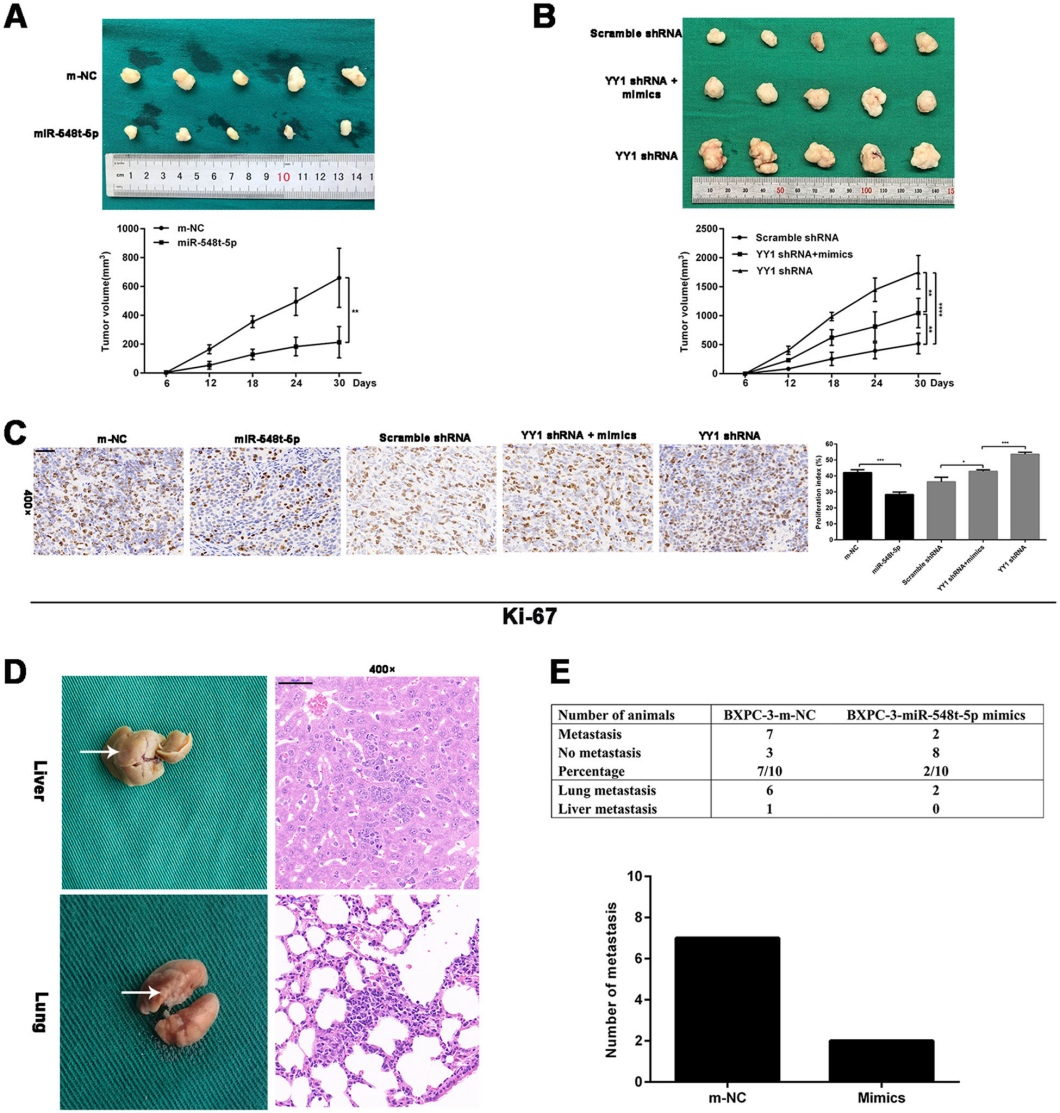
miR-548t-5p regulates the proliferation and metastasis of pancreatic cancer in vivo. **a** Tumors were obtained from nude mice injected subcutaneously with BXPC-3 cells transfected with miR-548t-5p-mimics lentiviruses or control lentiviruses and the tumor volume was made into growth curve. **b** Tumors were obtained from nude mice injected subcutaneously with BXPC-3 cells transfected with YY1 shRNA lentiviruses, control lentiviruses, and YY1 shRNA lentiviruses in combination with miR-548t-5p-mimics lentiviruses and the tumor volume was made into growth curve. **c** IHC detection and quantification of Ki-67 protein expression in subcutaneous tumors from mice injected with BXPC-3 cells. Scale bar is 50 μm. **d**, **e** Representative pictures of lung and liver metastases are presented. The table listed the incidence of metastases in the nude mice treated with miR-548t-5p-mimics lentiviral vector or controls. Scale bar is 50 μm. The data are presented as mean ± SD from three independent experiments. *P ≤ 0.05, **P ≤ 0.01, ***P ≤ 0.001, ****P ≤ 0.0001.

Stable cells (BXPC-3-YY1-shRNA + miR-548t-5p-mimics, BXPC-3-YY1-shRNA, BXPC-3-scrambled shRNA, BXPC-3-miR-548t-5p-mimics, and BXPC-3-m-NC) were implanted into BALB/cA-nu mice via subcutaneous or tail vein injection and we measured the sizes of the xenograft tumors every 6 days for 30 days. The tumor sizes formed by the BXPC-3-miR-548t-5p mimic cells were significantly smaller than those in the control group (Fig. 4a). The tumor sizes of the BXPC-3-YY1-shRNA group were significantly larger than those of the control group, whereas the tumor sizes of the BXPC-3-YY1-shRNA + miR-548t-5p-mimics group were smaller than those of the BXPC-3-YY1-shRNA group (Fig. 4b). Subsequently, Ki-67 staining revealed that miR-548t-5p overexpression resulted in a smaller proliferation index (Fig. 4c). In the tail vein metastasis model, metastases were observed in two of ten mice injected with BXPC-3-miR-548t-5p mimic cells; seven of the ten mice injected with control cells exhibited metastasis (Fig. 4d, h). However, there was no significant difference between the BXPC-3-miR-548t-5p-mimics group and the BXPC-3-m-NC group. The small number of experimental samples indicates that the experimental results need to be further confirmed. These in vivo study results indicate that miR-548t-5p is a functional target of YY1, and that miR-548t-5p inhibits the malignant biological behavior of PC in vivo.

### CXCL11 is a functional target of miR-548t-5p

**Fig. 5:**
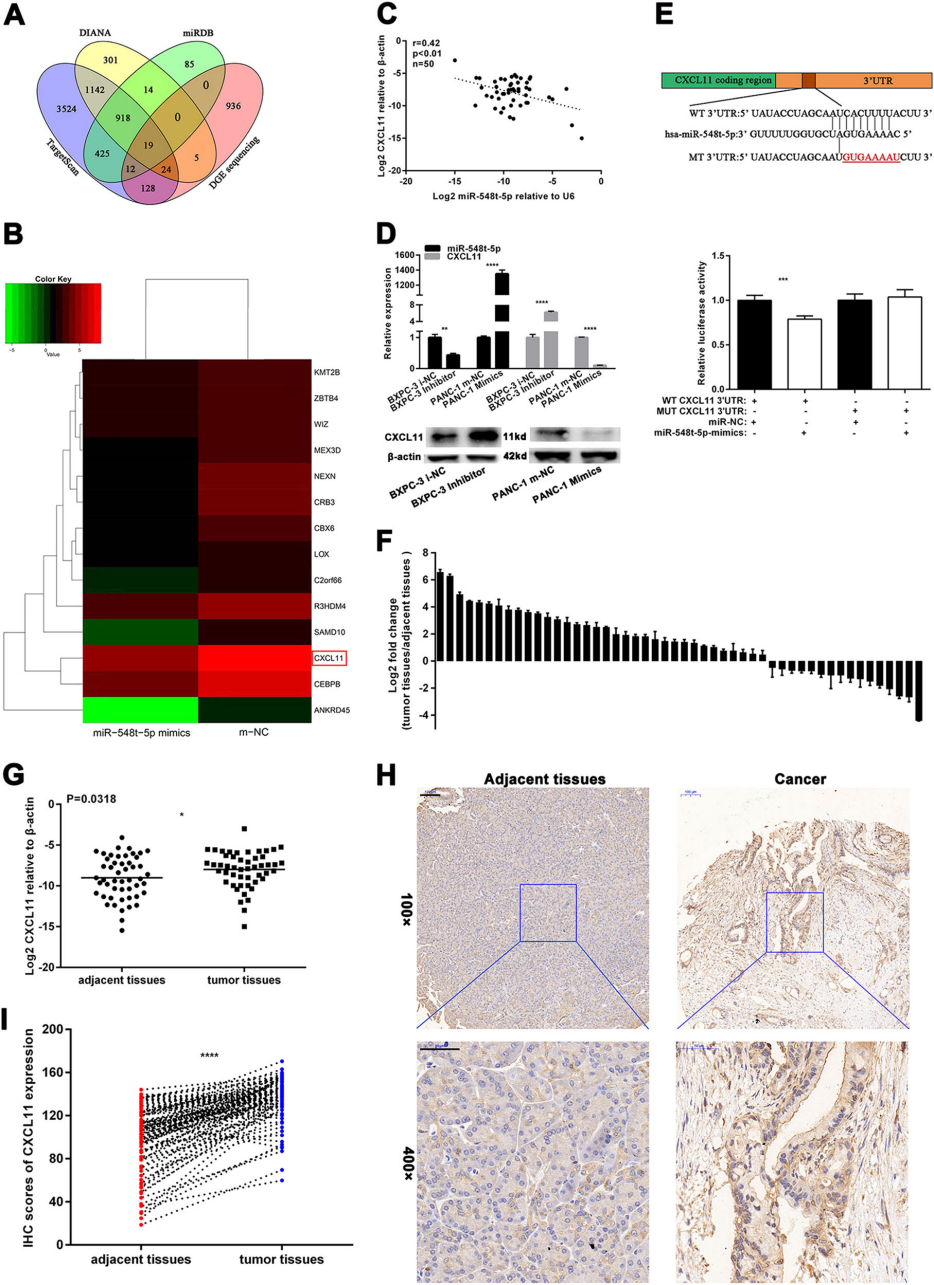
miR-548t-5p directly targets CXCL11 in PC. **a** The four-way Venn diagram indicates the numbers of genes that overlapped in three publicly available databases (DIANA, TargetScan, miRBD) and the DGE sequencing-based miR-548t-5p signature. **b** The heat map of the results of DGE sequencing based on 14 candidate genes that were downregulated in BXPC-3-miR-548t-5p-mimics cells. Red color represents an expression level above mean, green color represents an expression lower than the mean. **c** In human PC tissues, CXCL11 was positively correlated with miR-548t-5p expression. **d** miR-548t-5p and CXCL11 expression level in miR-548t-5p-mimics or miR-548t-5p inhibitor cells were measured by qRT-PCR. **e** Luciferase reporter assay was conducted to verify that miR-548t-5p directly bound to the 3′-UTR region of CXCL11. Luciferase activity was analyzed in cells cotransfected with miR-548t-5p-mimics or negative control with pGL3-CXCL11-WT or pGL3-CXCL11-MUT. **f** qRT-PCR analysis of CXCL11 expression in 50 pairs of human PC tissues and adjacent non-neoplastic tissues. **g** As analyzed by qRT-PCR, CXCL11 expression in PC tissues was significantly lower than that in the corresponding adjacent non-neoplastic tissues. **h** Representative IHC images of CXCL11 expression. Scale bar, 100 μm in ×100, 50 μm in ×400. **i** A histogram showing the IHC results of CXCL11 expression in the 95 pairs of human PC tissues and adjacent non-neoplastic tissues. The data are presented as mean ± SD from three independent experiments. *P ≤ 0.05, **P ≤ 0.01, ***P ≤ 0.001, ****P ≤ 0.0001.

To further explore the molecular mechanisms by which miR-548t-5p regulates PC cell biological behavior prognosis in vitro, three publicly available databases (miRDB, TargetScan, and DIANA) and DGE sequencing were employed to analyze target genes of miR-548t-5p, with comparison gene expression differences between miR-548t-5p overexpression and control conditions in the BXPC-3 cells achieved via DGE sequencing. An absolute value of the log2 ratio ≥ 1 was considered the threshold for judging the significance of gene expression, and the intersection of DGE sequencing and the three databases was used to determine target genes (Fig. 5a). The results showed an overlap of 19 genes; 14 genes, including *CXCL11*, were downregulated in the miR-548t-5p-overexpressing cells compared with the NC cells (Fig. 5b).

We then analyzed the expression of miR-548t-5p and CXCL11 in PC tissues by qRT-PCR. As shown in Fig. 5c, CXCL11 mRNA expression correlated negatively with miR-548t-5p expression (*p* < 0.01, *r* = 0.42). In addition, as measured by qRT-PCR and western blotting, miR-548t-5p knockdown led to increased CXCL11 expression in BXPC-3 cells and miR-548t-5p overexpression led to decreased CXCL11 expression in PANC-1 cells (Fig. 5d). Luciferase reporter assays were also performed, showing that CXCL11 is a direct target of miR-548t-5p (Fig. 5e).

### CXCL11 is upregulated in PC tissues and strengthens the malignant biological behavior of PC

The expression of CXCL11 in 50 pairs of human PC and adjacent non-neoplastic tissues was assessed by qRT-PCR. As illustrated in Fig. 5f, CXCL11 expression was upregulated in the tumor samples and it was significantly higher in PC tissues than in adjacent non-neoplastic tissues (Fig. 5g). Similarly, IHC was performed using TMAs consisting of 95 pairs of PC and adjacent tissues, and we found that CXCL11 expression was upregulated in PC tissues (Fig. 5h, i).

The correlation between CXCL11 expression and the clinicopathological characteristics of 50 PC patients was also analyzed; the median expression value was used as the cutoff value for low/high expression. As presented in Table 1, no significant association was observed regarding sex, age, tumor location, CA199, or CEA; however, CXCL11 expression did correlate positively with T staging, N staging, TNM staging, and nerve invasion (*p* = 0.004, *p* = 0.048, *p* = 0.045, and *p* = 0.024, respectively). The overall survival analysis showed no significant difference between high and low expression of CXCL11 according to the cutoff of the median expression value (Supplementary Fig. S3A; *p* = 0.1061). We also analyzed data in TCGA and the results revealed that patients with higher CXCL11 expression had shorter overall survival times (*p* = 0.034; Supplementary Fig. S3B).

### CXCL11 can partially restore the effect of miR-548t-5p on the proliferation and metastasis of PC cells

**Fig. 6:**
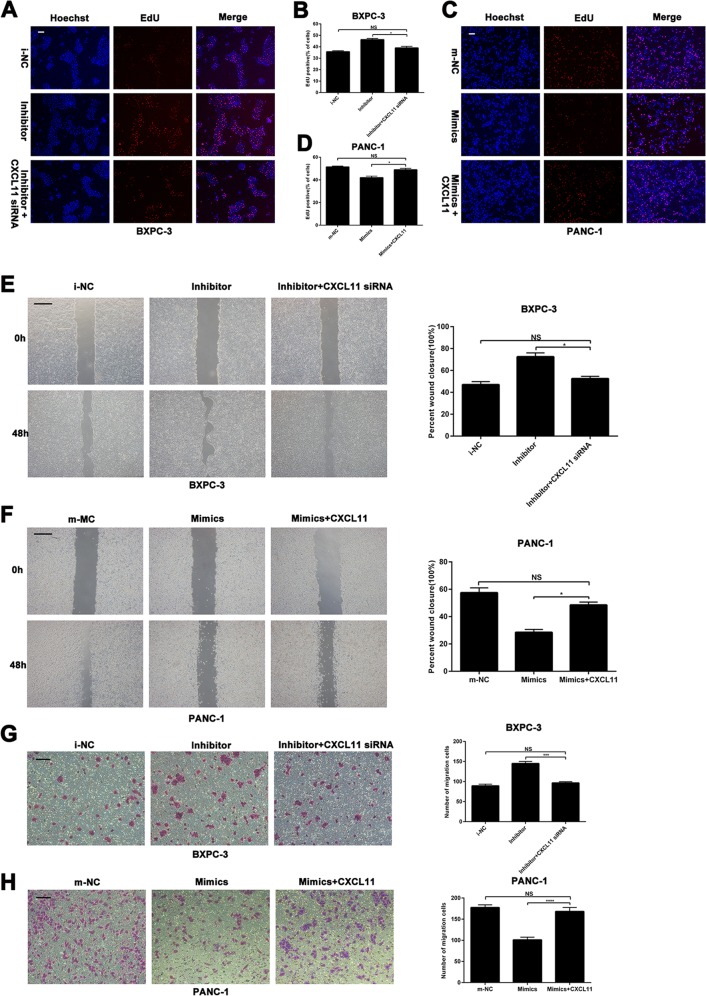
miR-548t-5p suppresses cell proliferation and metastasis by targeting CXCL11. **a**–**d** EdU assay was performed to analyze proliferation in BXPC-3-miR-548t-5p inhibitor cells transfected with CXCL11 siRNA and PANC-1-miR-548t-5p-mimics cells transfected with CXCL11-overexpression vector. Magnification, ×100; scale bar, 100 μm in **a** and **c**. **e**, **f** Wound-healing assays were performed to analyze migration in BXPC-3-miR-548t-5p inhibitor cells transfected with CXCL11 siRNA and PANC-1-miR-548t-5p-mimics cells transfected with CXCL11-overexpression vector. Magnification, ×100; scale bar, 100 μm. **g**, **h** Transwell assays were performed to analyze migration in BXPC-3-miR-548t-5p inhibitor cells transfected with CXCL11 siRNA and PANC-1-miR-548t-5p-mimics cells transfected with CXCL11-overexpression vector. Magnification, ×100; scale bar, 100 μm. The data are presented as mean ± SD from three independent experiments. *P ≤ 0.05, **P ≤ 0.01, ***P ≤ 0.001, ****P ≤ 0.0001.

To further investigate whether the effects of miR-548t-5p on PC cells are indeed mediated by CXCL11 in vitro, CXCL11 siRNA or an overexpression vector was used to knock down or overexpress CXCL11, respectively, in miR-548t-5p-knockdown or -overexpressing cells. The EdU assay indicated that the proliferation ability inhibited by miR-548t-5p overexpression was recovered by upregulating CXCL11, and that CXCL11 downregulation had the opposite effect on miR-548t-5p-knockdown cells (Fig. 6a–d). Similarly, the wound-healing and Transwell assays indicated that the migration ability inhibited by miR-548t-5p overexpression could partly be recovered by upregulating CXCL11, and that CXCL11 downregulation had the opposite effect on miR-548t-5p-knockdown cells (Fig. 6e–h). These results indicate that CXCL11, as a functional target of miR-548t-5p, may play an important role in inhibiting PC proliferation, migration, and invasion.

### CXCL11 promotes the proliferation of PC cells

**Fig. 7:**
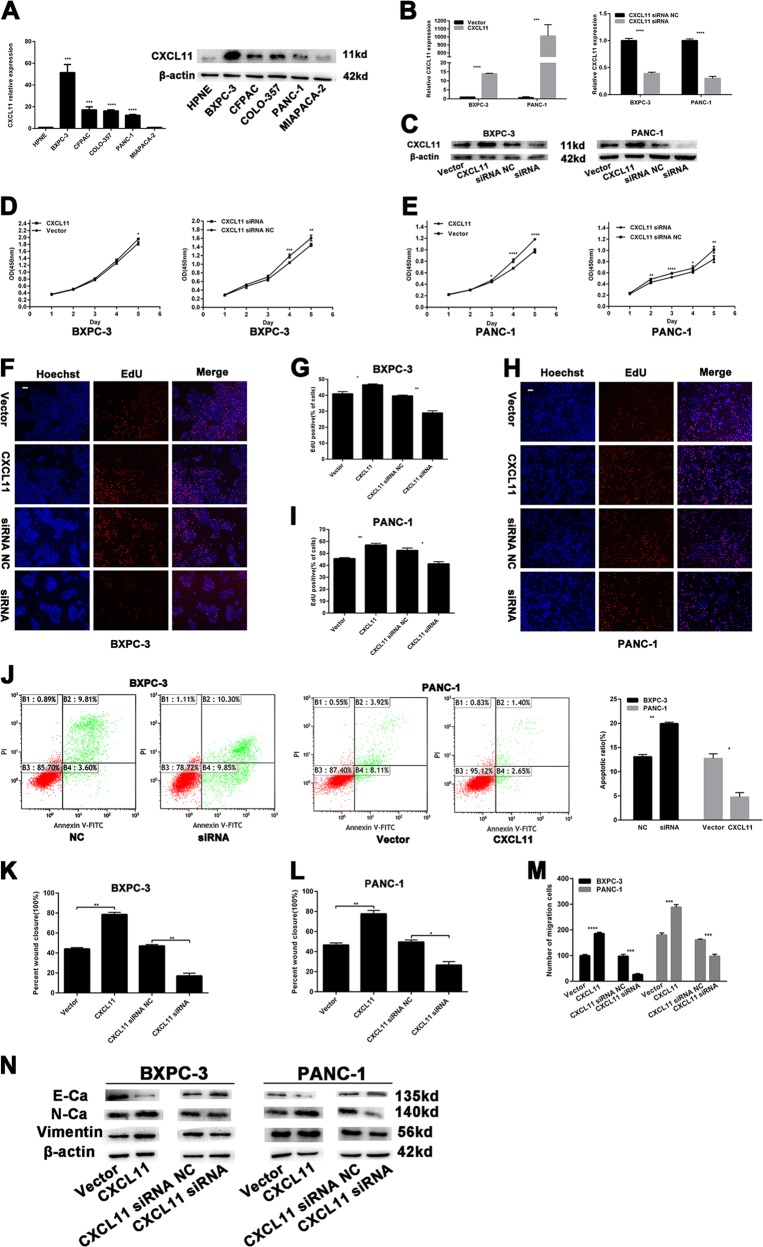
The effects of CXCL11 on PC cells proliferation and migration. **a** Relative expression of CXCL11 by qRT-PCR and western blotting in PC cell lines. **b**, **c** Relative expression of CXCL11 by qRT-PCR and western blotting in PC cells with CXCL11-overexpression vector and siRNA. **d**, **e** The CCK-8 assays were performed to analyze the effects of CXCL11 on cell proliferation. **f**–**i** EdU assays were performed to analyze the effects of CXCL11 on cell proliferation. Magnification, ×100; scale bar, 100 μm. **j** The flow cytometry analysis of the effect of CXCL11 expression alteration on cell apoptosis. **k**, **l** Wound-healing assays measured the effect CXCL11 on PC cell migration ability. **m** Transwell assays measured the effect CXCL11 on PC cell migration ability. **n** EMT-related protein was analyzed by western blotting assays in transfected PC cells. The data are presented as mean ± SD from three independent experiments. *P ≤ 0.05, **P ≤ 0.01, ***P ≤ 0.001, ****P ≤ 0.0001.

Figure 7a shows that CXCL11 expression was higher in most PC cell lines (CFPAC, COLO-357, BXPC-3, and PANC-1) than in HPNE cells, as assessed by qRT-PCR and western blotting. Thus, BXPC-3 and PANC-1 cells were transfected with the CXCL11-overexpression vector or siRNA; the transfection efficiency was assessed by miRNA qRT-PCR and western blotting (Fig. 7b, c), and CCK-8 and EdU assays were performed to evaluate the effect of CXCL11 on proliferation. CXCL11 overexpression significantly increased the OD450 values of BXPC-3 and PANC-1 cells compared with control cells, whereas CXCL11 knockdown had the opposite effects (Fig. 7d, e). In BXPC-3 and PANC-1 cells transfected with the CXCL11-overexpression vector, the proportion of EdU-positive nuclei was significantly increased and CXCL11 siRNA had the opposite effect (Fig. 7f-i).

Next, a FCM assay was performed and BXPC-3 cells transfected with CXCL11 siRNA exhibited a higher rate of apoptosis than the NC cells (Fig. 7j). In contrast, PANC-1 cells transfected with the CXCL11-overexpression vector had a lower rate of apoptosis than the NC cells. CXCL11 had no effect on the cell cycle (Supplementary Fig. S4A, B). The above results indicate that CXCL11 promotes PC cell proliferation.

### CXCL11 promotes metastasis of PC cells

As shown in Fig. 7k, l and Supplementary Fig. S5A, B, CXCL11 overexpression markedly promoted the migration of PANC-1 and BXPC-3 cells, and CXCL11-knockdown had the opposite effect. Similarly, more BXPC-3 and PANC-1 cells transfected with the CXCL11-overexpression vector migrated to the lower side of the membrane than the corresponding NC cells (Fig. 7m and and Supplementary Fig. S5C), whereas fewer cells transfected with CXCL11 siRNA migrated.

Moreover, western blotting was performed to examine the relationship between CXCL11 and EMT-related proteins. As depicted in Fig. 7n, N-cadherin and vimentin were downregulated after CXCL11 knockdown, whereas E-cadherin was upregulated. The opposite results were found in the CXCL11 overexpression cell lines. The above results reveal that CXCL11 promotes the metastasis of PC cells.

## Discussion

miRNA is a type of small molecular RNA with a length of ∼22 nucleotides that is highly conserved in evolution^18^. miRNAs bind completely or incompletely to target mRNAs to cause translation or degradation in the regulation of gene expression, affecting the biological characteristics of animals, plants and microorganisms^8,19,20^. In this study, we demonstrated a new molecular mechanism for the development of PC. miR-548t-5p was found to be downregulated in PC tissues and the YY1/miR-548t-5p/CXCL11 signaling axis regulates cell proliferation, migration, and invasion in human PC. These data suggest that miR-548t-5p and CXCL11 might serve as novel biomarkers or therapeutic targets for PC.

In our previous study, ChIP sequencing was performed with BXPC-3 cells to elucidate whether the YY1 gene can bind to genes that affect PC^7^. In the current study, miR-548t-5p was chosen as a potential target gene for YY1. miR-548t-5p is a less well-known gene and no one has concretely clarified its role in cancers. A previous study found high miR-548t expression in breast tumors, with a high recurrence score in 23 cases of early-stage breast carcinomas, but the number of cases was small and the molecular mechanism was not clarified^21^. In the present study, miR-548t-5p expression was significantly downregulated in PC tissues and its expression was closely related to tumor size, CA199, and neurological invasion. miRNA localization analysis is very important, because some miRNAs are located in the nucleus, which may yield effects opposite to those of miRNAs in the cytoplasm, and these molecules can have a positive regulatory effect by activating gene enhancers^22,23^. Therefore, a FISH assay was performed in this study, revealing that miR-548t-5p is located mainly in the cytoplasm. Through further in vitro and in vivo experiments, we found that miR-548t-5p can inhibit proliferation, promote apoptosis, and inhibit migration and invasion in PC cells. These results indicate that as a tumor suppressor, miR-548t-5p inhibits the tumorigenesis and development of PC and may be a potential target for the clinical treatment of this tumor.

YY1 is a multifunctional protein belonging to the GLI-Kruppel family that binds to other gene promoters mainly through its C-terminal zinc finger region^24,25^. YY1 is involved in the regulation of various genes that affect various biological functions of cells, including many miRNAs, such as miR-1260b^26,27^. The role YY1 plays as an oncogene or a tumor suppressor in cancers depends on the target gene that it regulates. When YY1 promotes the expression of oncogenes or downregulates that of antioncogenes, it induces tumorigenesis^28,29^. However, YY1 can also act as a tumor suppressor^30,31^. In our previous studies, we found that YY1 inhibits the proliferation, migration, and metastasis of PC^6,7^. Based on the ChIP sequencing results and the Jaspar database, we found that YY1 can bind to the promoter region of miR-548t-5p and we confirmed this using luciferase reporter and ChIP assays, which showed that YY1 has a positive regulatory effect on miR-548t-5p. Furthermore, we observed a positive correlation between YY1 and miR-548t-5p expression in 50 PC tissues, and BXPC-3 and PANC-1 cell lines through qRT-PCR. miR-548t-5p overexpression significantly restored the ability of YY1 knockdown to promote the proliferation and invasion of PC cells in both in vitro and in vivo experiments, and miR-548t-5p knockdown yielded the same results. Thus, YY1 promotes miR-548t-5p expression.

Chemokines play an important role in the interaction between tumor cells and their surrounding mesenchymal cells^32,33^. Indeed, chemokines enable tumor cells to migrate along the signal source of increasing chemokine concentration, thus playing an important role in tumor invasion and metastasis^34,35^. CXCL11 belongs to the ELRCXC chemokine family. Cancer cells cause CXCL11 secretion by regulating tumor stromal cells in the microenvironment^36,37^. CXCL11 not only regulates the targeted movement of cancer cells but is also involved in cancer cell entry into and out of blood vessels, immune evasion, proliferation, and angiogenesis^38^. CXCR3 and CXCR7 are CXCL11 receptors, and they are highly expressed in PC tissues; high CXCR3 and CXCR7 expression is associated with tumor apoptosis, invasion, and metastasis^39–41^. Nonetheless, the mechanism of CXCL11 in PC is still unclear. The findings of the present study reveal differential CXCL11 expression between PC tissues and paired adjacent tissues. CXCL11 expression was also found to be associated with nerve invasion, T staging, N staging and TNM staging. Although no significant difference was found in the Kaplan–Meier survival curve analysis of our 50 patients, significant differences were observed for patients in TCGA, which may be due to the sufficiently large sample size in the database. Furthermore, according to qRT-PCR results, the expressions of CXCL11 and miR-548t-5p in the 50 PC tissues and in BXPC-3 and PANC-1 cell lines were negatively correlated. CXCL11 overexpression also significantly restored the tumor suppressor role of miR-548t-5p. Thus, CXCL11 is a direct target of miR-548t-5p, as confirmed by the luciferase reporter assays.

It should be noted that PC is a highly invasive tumor and that the biological characteristics of neurotropic growth are common causes of recurrence and death in PC patients^42,43^. Accordingly, it is important to develop strategies to control the progression of PC by eliminating nerve invasion. In the current study, we found that the abnormal expression of miR-548t-5p and CXCL11 is related to the nerve infiltration of PC, which helps us better understand the relevant mechanism of nerve infiltration.

In summary, our study shows that miR-548t-5p, which is regulated by YY1, is significantly downregulated in PC and suppresses tumor growth and metastasis by targeting CXCL11. Furthermore, miR-548t-5p and CXCL11 expression is closely related to the clinicopathology of PC patients. Therefore, our results suggest that targeting the YY1/miR-548t-5p/CXCL11 axis may be a potent therapeutic approach for PC.

## Supplementary information

Supplementary figure legends

FIG S1

FIG S2

FIG S3

FIG S4

FIG S5

Table. S1
