# Editorial Expression of Concern to: The YY1/miR-548t-5p/CXCL11 signaling axis regulates cell proliferation and metastasis in human pancreatic cancer

**DOI:** 10.1038/s41419-025-07740-8

**Published:** 2025-05-23

**Authors:** Wan-Li Ge, Qun Chen, Ling-Dong Meng, Xu-Min Huang, Guo-dong Shi, Qing-Qing Zong, Peng Shen, Yi-Chao Lu, Yi-Han Zhang, Yi Miao, Jing-Jing Zhang, Kui-Rong Jiang

**Affiliations:** 1 Pancreas Center, The First Affiliated Hospital of Nanjing Medical University, Nanjing, China; 2 Pancreas Institute, Nanjing Medical University, Nanjing, China; 3 Ultrasonography, The First Affiliated Hospital of Nanjing Medical University, Nanjing, China

Editorial Expression of Concern to: *Cell Death and Disease* 10.1038/s41419-020-2475-3, published online 27 April 2020

The Editors would like to alert the readers that concerns have been raised regarding data similarity between Fig. 2C PANC-1 Mimics image in this article and Fig. 2E MiaPaCa ROBO-1 image in a later article from the same author group [1]. Readers are therefore advised to interpret these data with caution.

Wan-Li Ge, Qun Chen, Ling-Dong Meng, Guo-dong Shi, Peng Shen, Yi-Han Zhang, Yi Miao, Jing-Jing Zhang and Kui-Rong Jiang agree to this Editorial Expression of Concern.

Xu-Min Huang, Qing-Qing Zong and Yi-Chao Lu have not responded to any correspondence from the editor or publisher about this Editorial Expression of Concern.
