# Supplementary material for: The YY1/miR-548t-5p/CXCL11 signaling axis regulates cell proliferation and metastasis in human pancreatic cancer
Source: Cell Death Dis. 2020 Apr 27;11(4):294. doi: 10.1038/s41419-020-2475-3 (PMC7186231; doi:10.1038/s41419-020-2475-3)
Supplement: Supplementary file 1 — Supplementary figure legends [file 41419_2020_2475_MOESM1_ESM.docx]

**Figure S1** The effect of miR-548t-5p on cell apoptosis of PC cells.

**A** MicroRNA FISH showed that miR-548t-5p was mostly located in the cytoplasm. Magnification, 400 ×; scale bar, 50 μm. **B** The flow cytometry analysis of the effect of miR-548t-5p expression alteration on cell apoptosis. The data is presented as mean ± SD (standard deviation) from three independent experiments. *P ≤ 0.05, **P ≤ 0.01, ***P ≤ 0.001, ****P ≤ 0.0001.

**Figure S2** The effects of miR-548t-5p on PC cells migration and invasion.

**A- B** Wound healing assays measured the effect of miR-548t-5p on PC cell migration ability. Magnification, 100×; scale bar, 100 μm. **C** Transwell assays measured the effect of miR-548t-5p on PC cell invasion ability. Magnification, 100 ×; scale bar, 100 μm.

**Figure S3** Kaplan-Meier curves for overall survival (OS) by CXCL11 expression.

**A** Kaplan-Meier curves of 50 patients in current study for overall survival (OS) by CXCL11 expression. **B** Kaplan-Meier curves of 162 patients from TGCA database for overall survival (OS) by CXCL11 expression.

**Figure S4** Flow cytometry analysis of the effects of CXCL11 on cell apoptosis and cycle.

**A-B** No significant difference was found on flow cytometry analysis of the effect of CXCL11 expression alteration on cell apoptosis cell cycle status. The data is presented as mean ± SD (standard deviation) from three independent experiments. *P ≤ 0.05, **P ≤ 0.01, ***P ≤ 0.001, ****P ≤ 0.0001.

**Figure S5** The effects of CXCL11 on PC cells migration.

**A-B** Wound healing assays measured the effect CXCL11 on PC cell migration ability. Magnification, 100×; scale bar, 100 μm. **C** Transwell assays measured the effect CXCL11 on PC cell migration ability. Magnification, 100×; scale bar, 100 μm.
