# Supplementary figures and images for: The YY1/miR-548t-5p/CXCL11 signaling axis regulates cell proliferation and metastasis in human pancreatic cancer
Source: Cell Death Dis. 2020 Apr 27;11(4):294. doi: 10.1038/s41419-020-2475-3 (PMC7186231; doi:10.1038/s41419-020-2475-3)

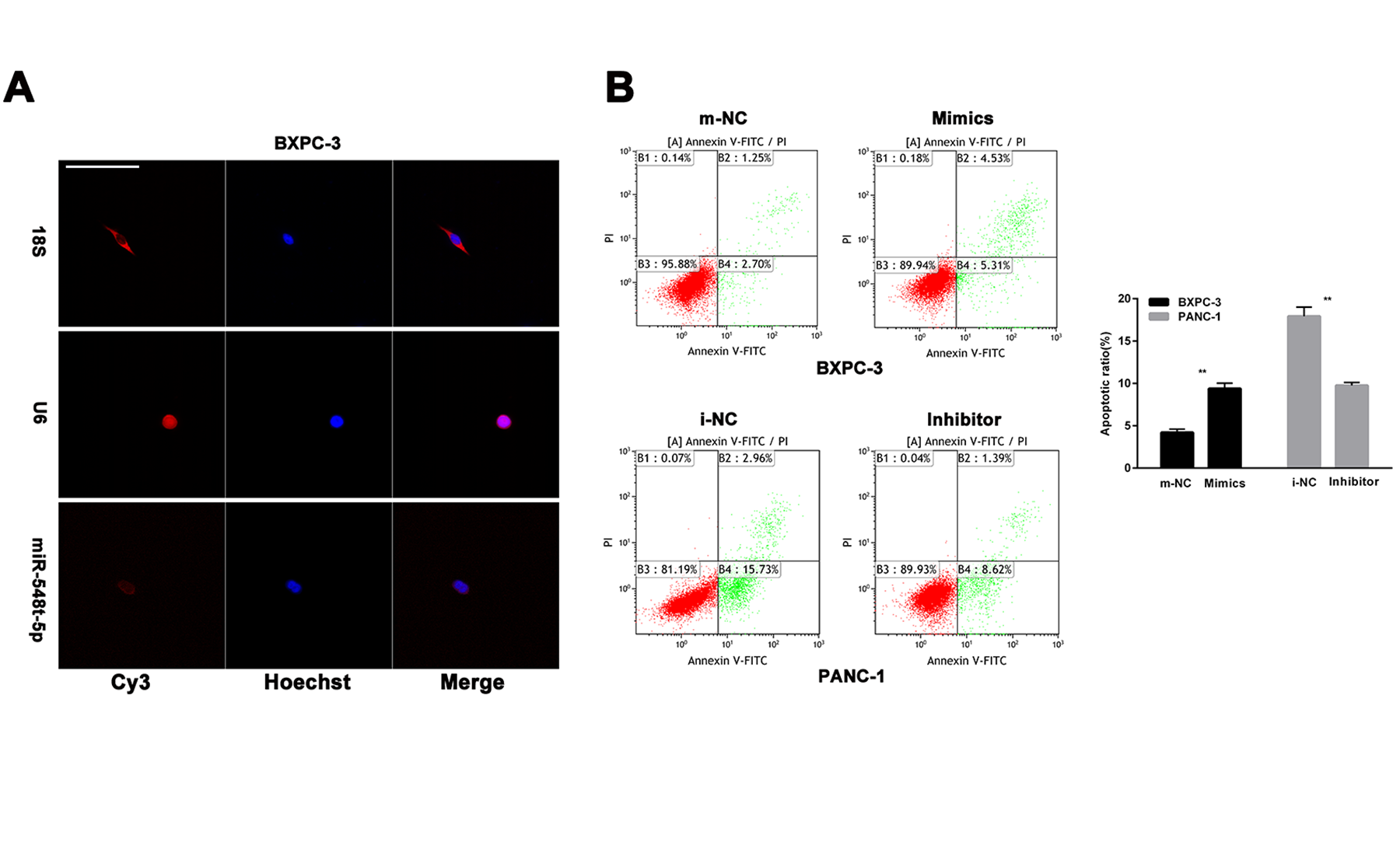

Supplement: Supplementary file 2 — FIG S1 [file 41419_2020_2475_MOESM2_ESM.tif]

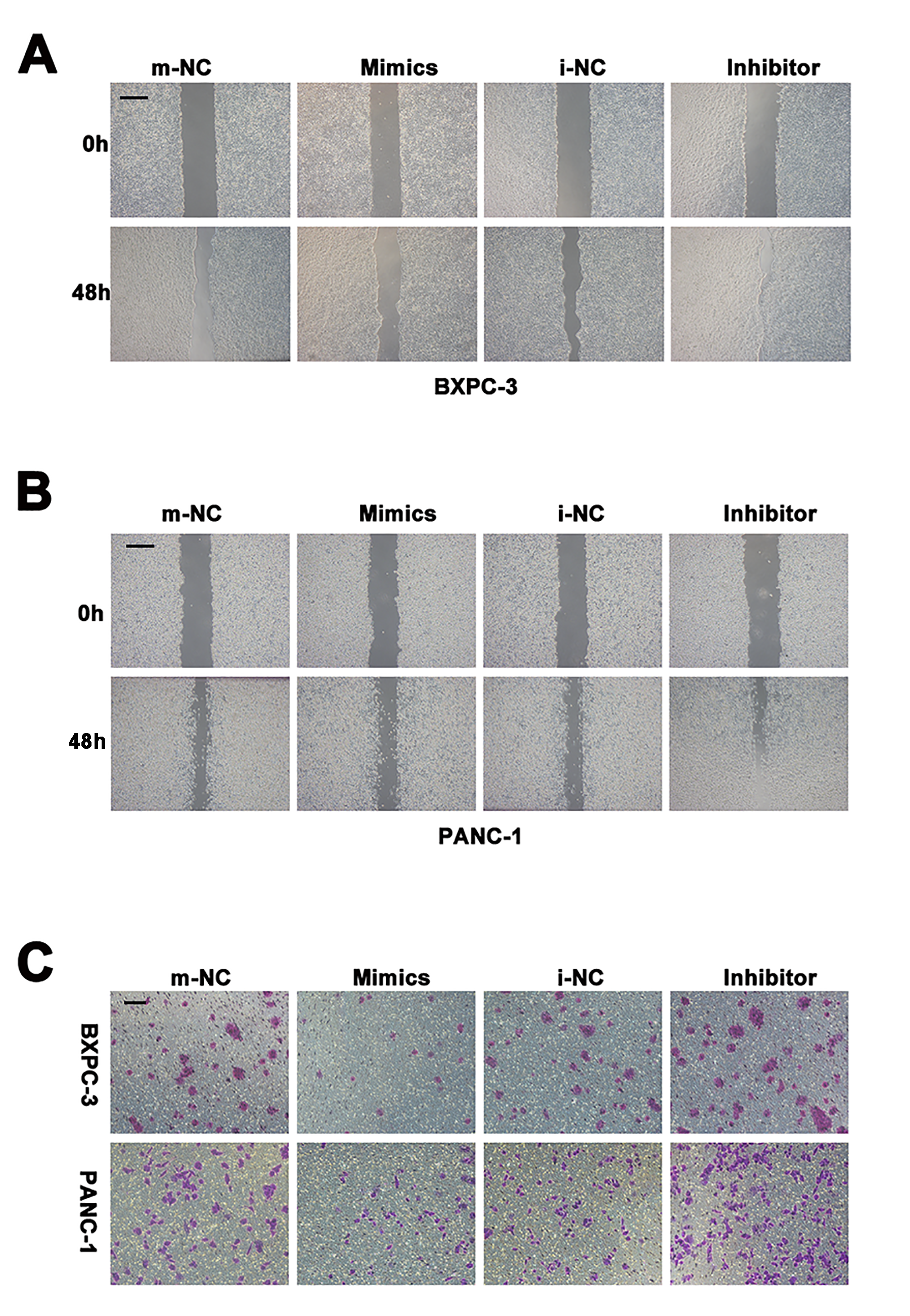

Supplement: Supplementary file 3 — FIG S2 [file 41419_2020_2475_MOESM3_ESM.tif]

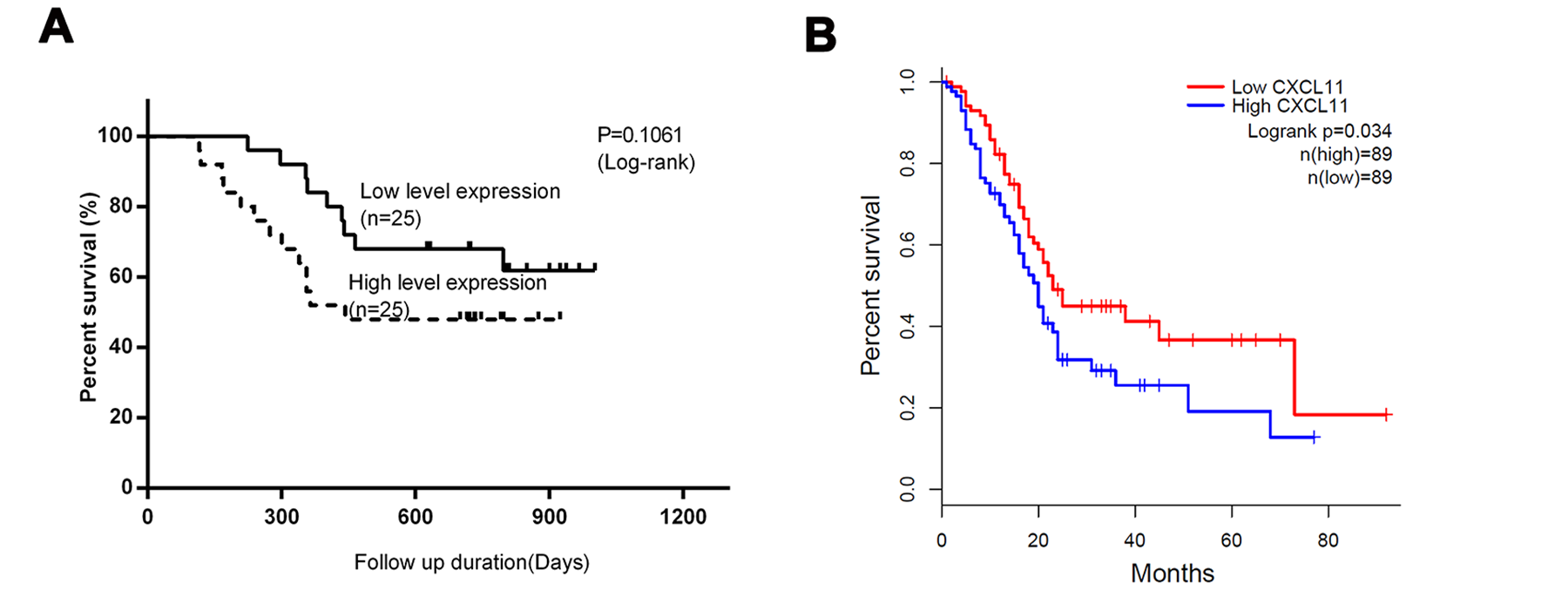

Supplement: Supplementary file 4 — FIG S3 [file 41419_2020_2475_MOESM4_ESM.tif]

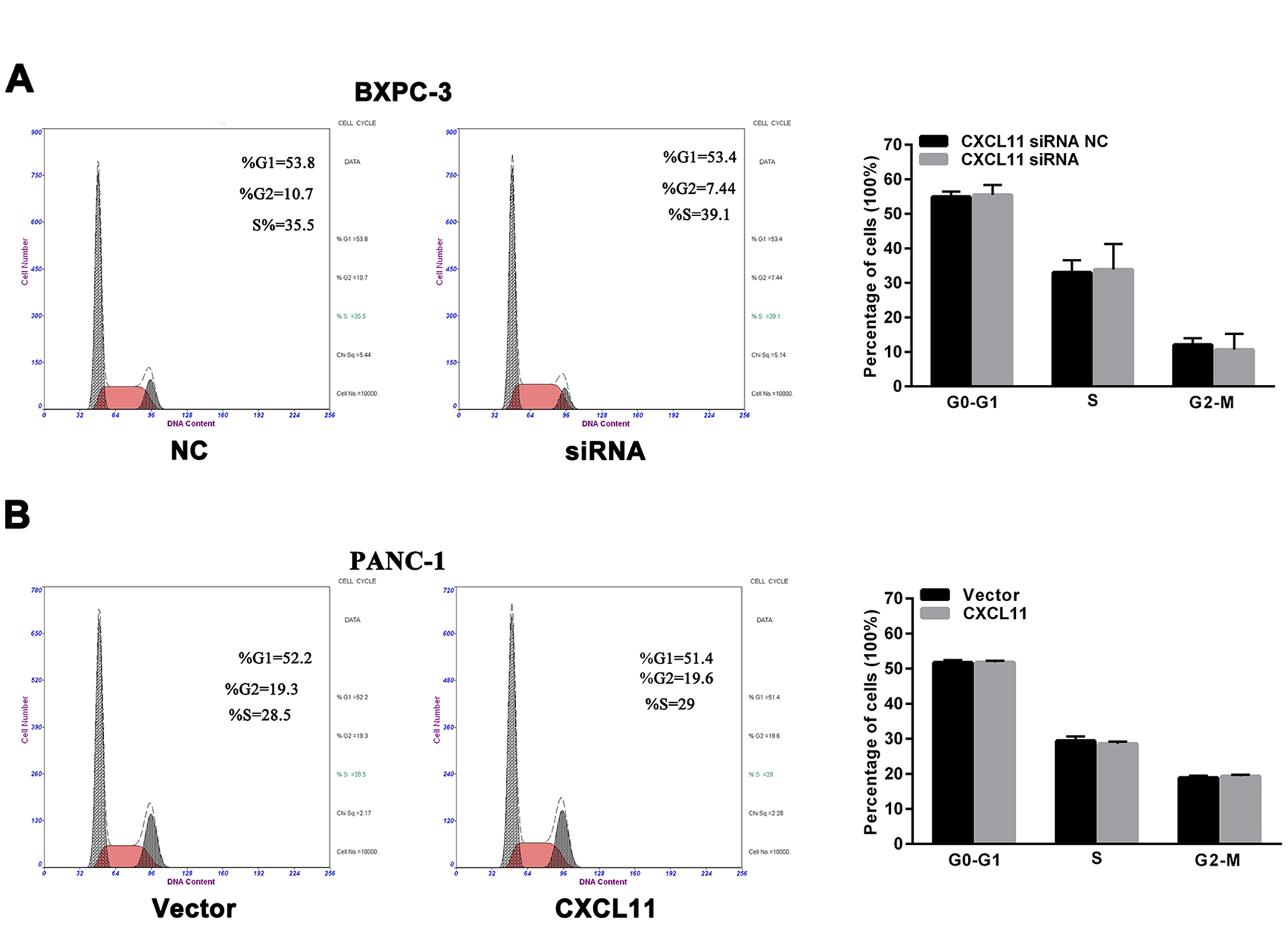

Supplement: Supplementary file 5 — FIG S4 [file 41419_2020_2475_MOESM5_ESM.tif]

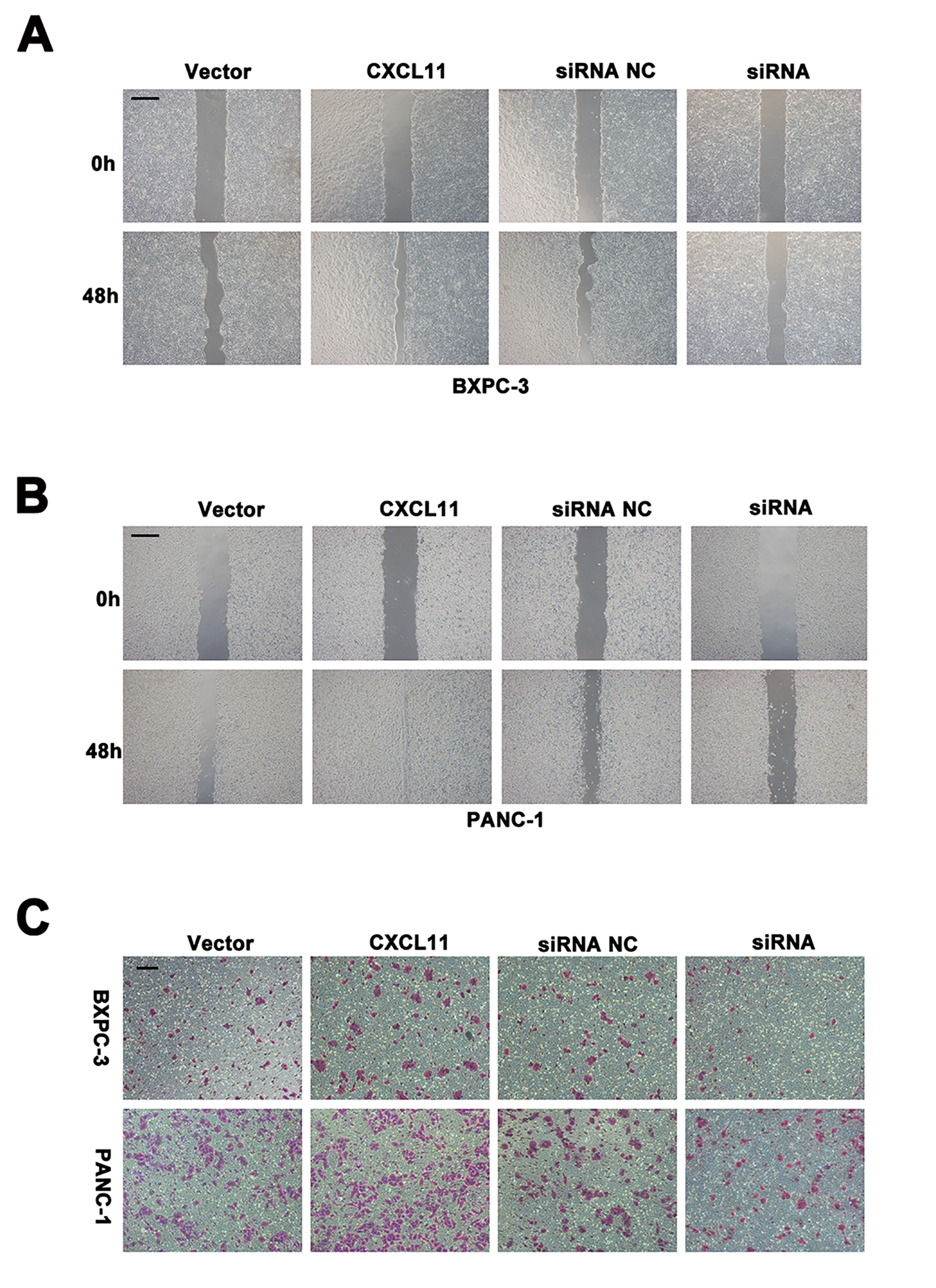

Supplement: Supplementary file 6 — FIG S5 [file 41419_2020_2475_MOESM6_ESM.tif]
