# Supplementary material for: The YY1/miR-548t-5p/CXCL11 signaling axis regulates cell proliferation and metastasis in human pancreatic cancer
Source: Cell Death Dis. 2020 Apr 27;11(4):294. doi: 10.1038/s41419-020-2475-3 (PMC7186231; doi:10.1038/s41419-020-2475-3)
Supplement: Supplementary file 7 — Table. S1 [file 41419_2020_2475_MOESM7_ESM.docx]

**Table S1** List of the oligonucleotides primers used for amplification

| Genes | Primer sequence(5’-3’) |
| --- | --- |
| miR-548t-5p | F: 5’- CAAAAGTGATCGTGGTTTTTG-3’ |
|  | R: 5’- GCGAGCACAGAATTAATACGAC-3’ |
| CXCL11: | F: 5’- GACGCTGTCTTTGCATAGGC-3’ |
|  | R: 5’- GGATTTAGGCATCGTTGTCCTTT-3’ |
| U6 | F: 5’- CTCGCTTCGGCAGCACA-3’ |
|  | R: 5’- AACGCTTCACGAATTTGCGT-3’ |
| β-actin | F: 5’- AGCGAGCATCCCCCAAAGTT-3’ |
|  | R: 5’- GGGCACGAAGGCTCATCATT-3’ |
| YY1 | F: 5’- ACGGCTTCGAGGATCAGATTC-3’ |
|  | R: 5’- TGACCAGCGTTTGTTCAATGT-3’ |
| 18S RNA | F: 5’- CAGCCACCCGAGATTGAGCA-3’ |
|  | R: 5’- TAGTAGCGACGGGCGGTGTG-3’ |
